# FOVOCIP study: a multicenter randomized trial of fosfomycin versus ciprofloxacin for febrile neutropenia in hematologic patients—efficacy and microbiologic safety

**DOI:** 10.1186/s13063-023-07702-5

**Published:** 2023-10-27

**Authors:** Ainhoa Fernández Moreno, Lucía Lavín-Alconero, Paula López de Ugarriza, Laura Solán Blanco, Sara Cáceres Hernández, Juan Miguel Bergua Burgués, María Izquierdo de Miguel, Ana Julia González Huerta, Marta Polo Zarzuela, Blanca Boluda, Karem Humala, Maria Luisa Calabuig, Maria Luz Amigo, Marián Cuesta Casas, María del Mar García-Saiz, Ana Fernández Verdugo, Javier Fernández Domínguez, Teresa Bernal

**Affiliations:** 1 Hematology Department, University Hospital Central of Asturias, Avenida Roma, 33011 Oviedo, Spain; 2 Instituto de Investigación Sanitaria del Principado de Asturias, Avenida Roma, 33011 Oviedo, Spain; 3 Instituto Universitario de Oncología del Principado de Asturias, C/ Fernando Bongera, 33006 Oviedo, Spain; 4 Clinical Trials Agency Valdecilla-IDIVAL, Marqués de Valdecilla University Hospital, Av. Valdecilla, 25, 39008 Santander, Cantabria Spain; 5 Department of Clinical Pharmacology, Marqués de, Valdecilla University Hospital, Av. Valdecilla, 25, 39008 Santander, Cantabria Spain; 6 Hematology Department, Fundación Jiménez Díaz, Av. Reyes Católicos, 28040 Madrid, Spain; 7 Hematology Department, San Pedro Alcántara Hospital, C/Pablo Naranjo Porras, 10003 Cáceres, Spain; 8 Hematology Department, University Clinic Hospital San Carlos, C/Prof. Martín Lagos, 28040 Madrid, Spain; 9 Hematology Department, Instituto de Investigación, University Hospital La Fe, Avinguda Fernando Abril Martorell, 46026 Valencia, Spain; 10 Hematology Department, University Hospital La Paz, Paseo de La Castellana, 28046 Madrid, Spain; 11 Hematology Department, University Clinic Hospital of Valencia, Av Blasco Ibañez, 46010 Valencia, Spain; 12 Hematology Department, University Hospital Morales Messeguer, C/Marqués de los Vélez, 30008 Murcia, Spain; 13 Hematology Department, University Hospital Carlos Haya, Av Carlos Haya, 29010 Malaga, Spain; 14 Microbiology Department, University Hospital Central of Asturias, Avenida Roma, 33011 Oviedo, Spain; 15 CIBER_Enfermedades Respiratorias ISCIII, Av. Monforte de Lemos, 3-5, Pabellón 11, Planta 0, 28029 Madrid, Spain

**Keywords:** Hematological neoplasms, Febrile neutropenia, Prophylaxis, Gram-negative resistant bacteria, Randomized controlled trial

## Abstract

**Background:**

Multidrug-resistant Gram-negative bacterial (MRGNB) infections represent a major public health threat. Cancer patients and, among them, hematological patients are most vulnerable to these infections. Gut colonization by MRGNB is a common phenomenon occurring during hospitalization and chemotherapy exposure. In the neutropenic phase that occurs after chemotherapy, MRGNB translocation occurs increasing patient’s mortality. Fluoroquinolone prophylaxis with ciprofloxacin or levofloxacin efficacy is now being questioned due to the increase of incidence in MRGNB.

**Methods:**

A phase III randomized, controlled, clinical trial, open-label parallel-group with a 1:1 ratio, aimed to demonstrate the non-inferiority of oral fosfomycin versus oral ciprofloxacin for febrile neutropenia prevention in patients with acute leukemia (AL) or hematopoietic cell transplant (HSC) receptors. Weekly surveillance cultures are planned to detect gut colonization. Changes in fecal microbiome at the beginning and end of prophylaxis will also be analyzed.

**Discussion:**

This trial will provide evidence of the efficacy of an alternative drug to ciprofloxacin for febrile neutropenia prevention in high-risk hematological patients. The battery of planned microbiological studies will allow us to evaluate prospectively the microbiological safety of both pharmacological strategies in terms of the selection of MRGNB occurring in each arm. In addition, valuable information on the way in which each drug changes the fecal microbiome of the patients throughout the treatment will be generated.

**Trial registration:**

Clinical trials NCT05311254, Registered on 5 April 2022, https://clinicaltrials.gov/ct2/show/NCT05311254?term=FOVOCIP&cntry=ES&draw=2&rank=1. Protocol version: 3.0, dated 20 May 2022.

## Administrative information

Note: the numbers in curly brackets in this protocol refer to SPIRIT checklist item numbers. The order of the items has been modified to group similar items (see http://www.equator-network.org/reporting-guidelines/spirit-2013-statement-defining-standard-protocol-items-for-clinical-trials/).

**Table:** 

Title {1}	FOVOCIP: a multicenter randomized trial of fosfomycin versus ciprofloxacin for febrile neutropenia in hematologic patients—efficacy and microbiologic safety.
Trial registration {2a and 2b}.	Clinical trials NCT05311254, Registered on 5 April 2022, https://clinicaltrials.gov/ct2/show/NCT05311254?term=FOVOCIP&cntry=ES&draw=2&rank=1
Protocol version {3}	Version 3.0 20 of May 2022
Funding {4}	This study is a non-commercial study funded by the national aid called PI21—Health Research Projects (AES 2021). Modality health research projects has been received for the development of the following clinical trial.
Author details {5a}	1. Hematology Department, University Hospital Central of Asturias. Avenida Roma, 33011, Oviedo, Spain 2. Instituto de Investigación Sanitaria del Principado de Asturias. Avenida Roma, 33011, Oviedo, Spain. 3. Instituto Universitario de Oncología del Principado de Asturias. C/ Fernando Bongera, 33006 Oviedo, Spain. 4. Clinical Trials Agency Valdecilla-IDIVAL, Marqués de Valdecilla University Hospital, Av. Valdecilla, 25, 39008 Santander, Cantabria, Spain 5. Department of Clinical Pharmacology, Marqués de Valdecilla University Hospital, Av. Valdecilla, 25, 39008 Santander, Cantabria, Spain. 6. Hematology Department, Fundación Jiménez Díaz. Av. Reyes Católicos, 28040, Madrid, Spain. 7. Hematology Department, San Pedro Alcántara Hospital. C/ Pablo Naranjo Porras, 10003, Cáceres, Spain. 8. Hematology Department, University Clinic Hospital San Carlos. C/ Prof. Martín Lagos, 28040, Madrid. 9. Hematology Department, Instituto de Investigación, University Hospital La Fe. Avinguda Fernando Abril Martorell, 46026. 10. Hematology Department, University Hospital La Paz. Paseo de la Castellana, 28046, Madrid, Spain. 11. Hematology Department, University Clinic Hospital of Valencia. Av Blasco Ibañez, 46010, Valencia. 12. Hematology Department, University Hospital Morales Messeguer. C/ Marqués de los Vélez, 30008, Murcia. 13. Hematology Department, University Hospital Carlos Haya. Av Carlos Haya, 29010, Malaga, Spain. 14. Microbiology Department, University Hospital Central of Asturias. Avenida Roma, 33011, Oviedo, Spain. 15. CIBER_Enfermedades Respiratorias ISCIII. Av. Monforte de Lemos, 3–5. Pabellón 11. Planta 0 28029 Madrid
Name and contact information for the trial sponsor {5b}	Fundación para la Investigación y la innovación (FINBA), Instituto de Investigación sanitaria (ISPA) Faustino Blanco as representative of the sponsor comunicacion@finba.es
Role of sponsor {5c}	The authors declare that they have no competing interests. The study was designed and conceived independently from the funding and sponsor institutions, neither of which had a role in the collection, management, analysis or interpretation of data or the writing of the final manuscript.

## Introduction

### Background and rationale {6a}

The use of fluoroquinolones (FQ) ciprofloxacin or levofloxacin is an established practice for the prevention of infectious events in hematologic patients in whom long lasting and profound neutropenia (absolute neutropenia count ≤ 0.1/ × 10^9^/L for more than 7 days) is expected. Patients included in this category are acute leukemia (AL) patients treated with intensive chemotherapy and allogeneic hematopoietic stem cell transplant (HSCT) recipients [1, 2]. In these patients, FQ exposure is associated with the development of multidrug-resistant Gram-negative bacteria (MRGNB) gut colonization [3]. These infections are associated with increased mortality in HSCT receptors and AL patients [4, 5]. A way of limiting the spread of MRGNB is by means of systematic surveillance of gut colonization by these bacteria. This practice has shown to prevent nosocomial dissemination by carbapenemase-producing enterobacteria [6]. In hematological patients, systematic surveillance cultures are being increasingly performed because they also provide valuable information for adjusting antimicrobial therapy at the time of febrile neutropenia [7, 8].

The concerns about the increasing incidence of MRGNB and its risks have led to questioning the actual recommendation of FQ prophylaxis, especially in those settings with a baseline resistance rate above 20% [9]. However, it should be noted that no controlled clinical trial has supported abandoning prophylaxis. Moreover, leaving the decision of FQ prophylaxis to physician’s criteria puts the patients at risk of not receiving an adequate treatment.

In addition to multidrug resistance associated to FQ, musculoskeletal and neurological adverse events (AEs) related to them represent another problem. For this reason, the European Pharmacovigilance Risk Assessment Committee recommends restricting their use (https://www.aemps.gob.es/informa/notasinformativas/medicamentosusohumano-3/seguridad-1/2018/ni_muh_fv-14-2018-quinolonas-fluoroquinolonas/).

Finally, another aspect for which FQ may adversely impact the outcome of patients is the modification of the intestinal microbiota. It has been demonstrated that replacement of the normal fecal microbiota with an abnormal one occurs in patients undergoing allogeneic HSCT [10]. Among other factors, this event is the consequence of the exposure to antimicrobials, but it is also influenced by the occurrence of graft-versus-host disease. The available evidence is still insufficient, since other factors such as immunomodulatory therapies, types of transplantation (autologous versus allogeneic), chemotherapy, and even the state of the neoplasm (active versus remission) should be analyzed. Furthermore, it has also been documented that the intestinal microbiota is related to the development of infections in patients with AL, showing that those patients with low α-diversity have a higher risk of developing them [11].

All the aforementioned limitations related to FQ prophylaxis have pointed to the urgent need for exploring new prophylactic drugs. In the past decades, other drugs were evaluated without positive results. This is the case of cephalosporins [12] or cotrimoxazole, which was abandoned due to myelotoxicity and lack of activity against *P. aeruginosa* [13].

Fosfomycin is a broad-spectrum bactericidal antibiotic approved for the treatment of uncomplicated urinary tract infections, gastrointestinal tract infections, and dermatological infections caused by microorganisms sensitive to this antibiotic. It has activity against Gram-positive and Gram-negative microorganisms and against extended-spectrum B-lactamase *Enterobacteriaceae* (ESBL) and/or carbapenemase-producing strains of enterobacteria and multidrug-resistant *P. aeruginosa*, which makes its use attractive even in endemic settings [14].

In view of the above, the need of conducting this study is justified. The results could demonstrate the non-inferiority of oral fosfomycin compared to oral ciprofloxacin in preventing infections in patients with neutropenia at high risk of developing infection as well as its effect on the intestinal flora as assessed by surveillance cultures and fecal microbiota analysis.

### Choice of comparator {6b}

The choice of ciprofloxacin over levofloxacin is based on the evidence supporting its superiority in the prevention of febrile neutropenia [2] and the extensive experience accumulated with its use over two decades in this setting.

### Objectives {7}

#### Hypotheses

The use of oral fosfomycin is not inferior to oral ciprofloxacin in the prevention of fever of infectious origin in high-risk neutropenic patients. This hypothesis is based not only on the broad-spectrum activity of fosfomycin but also on the low rate of resistance to this drug. In addition, we anticipate that the clinical efficacy of fosfomycin will be accompanied by an acceptable safety profile. Thus, there is potential for fosfomycin to be useful as a prophylactic agent in settings endemic for GNMRB.

#### Objective

The main objective of this clinical trial is to demonstrate non-inferiority in efficacy of oral fosfomycin versus oral ciprofloxacin in preventing febrile neutropenia in patients with acute leukemia treated with intensive chemotherapy and/or recipients of hematopoietic stem cell transplantation.

### Trial design {8}

The FOVOCIP study is a randomized, open-label, phase III clinical trial with a non-inferiority design, in which patients are randomized in a 1:1 ratio into two parallel, controlled prophylaxis groups: fosfomycin or ciprofloxacin. The groups are stratified by site and by underlying disease (acute leukemia or stem cell transplantation).

FOVOCIP is a pragmatic study and very well adapted to the usual clinical practice of the sites of the public health system in Spain.

## Methods: participants, interventions, and outcomes

### Study setting {9}

The sponsor of this study is FINBA and plans to include 156 patients over 2 years; therefore, 11 Spanish national hospitals are needed to achieve the described objective.

The hospitals included are:

- ✓ University Hospital Central of Asturias.
- ✓ University Hospital La Paz.
- ✓ University Clinic Hospital San Carlos.
- ✓ Hospital San Pedro of Alcántara.
- ✓ University Hospital Morales Messeguer.
- ✓ University Hospital La Fe.
- ✓ University Clinic Hospital of Valencia.
- ✓ University Hospital of Málaga.
- ✓ University Hospital Fundación Jiménez Díaz.
- ✓ University Clinic Hospital Lozano Blesa.
- ✓ University Hospital of Burgos.
- ✓ These are academic hospital centers with experience in the management of acute leukemias and stem cell transplants. They are located around several regions in Spain. Therefore, they represent a broad spectrum of our country geography. This guarantees the representativeness of the Spanish population and the achievement of the planned recruitment.
- ✓ The Clinical Trials agency and SCReN (Spanish Clinical Research Network) were responsible for obtaining authorization from the local ethics committee (EC) and notifying to the Spanish Agency of Medicines and Medical Devices of the start-up of the study.

### Eligibility criteria {10}

Candidate selection will be performed in 2 different settings depending on the therapeutic procedure:

- In subjects with a suspected diagnosis of acute leukemia, where the diagnosis of the neoplasm is made as an inpatient, the selection will take place once the diagnosis has been confirmed and prior to the start of intensive chemotherapy.
- In HSCT recipients, selection will take place on an outpatient basis during the pre-transplant evaluation.

**Inclusion criteria**

Subjects must meet all of the following inclusion criteria:

1. Subjects must be able to understand the study procedures, comply with them, and give written informed consent prior to any specific study procedure.
2. Adult subjects 18 years of age who meet one of the following criteria:
  Diagnosis of acute leukemia who are to receive a first course of intensive chemotherapy or
  Candidates to receive a first allogeneic hematopoietic progenitor transplant with myeloablative conditioning or
  Candidates to receive a first allogeneic hematopoietic progenitor transplant with reduced-intensity conditioning* or an autologous hematopoietic progenitor transplant provided that at least one of the following risk factors for infection is present:
    -  Performance status (Eastern Cooperative Oncology Group, ECOG) ≥ 2.
    -  Expected grade 3–4 mucositis.
    -  Age ≥ 65 years.
    -  Hematopoietic Comorbidity Transplant Index (HCTI) ≥ 3.
    -  Serum albumin < 35 g/L.
    -  Active or refractory neoplasia at the time of stem cell transplantation.
    -  Total dose of etoposide > 500 mg/m.^2^
    -  Total dose of cytarabine > 1 g/m.^2^

3. Performance status (Eastern Cooperative Oncology Group, ECOG) 0 to 3.
4. Adequate organ function defined as:
  Liver: bilirubin, alkaline phosphatase, or SGOT < 3 times the upper limit of normal (unless attributable to tumor activity)
  Renal: creatinine ≤ 250 μmol/l (2.5 mg/dL) (unless attributable to leukemic infiltration)

5. Life expectancy greater than 3 months
6. Women of childbearing age must not be pregnant or breastfeeding and must have a negative pregnancy test at the time of screening. Women of childbearing age and men with female partners of childbearing age must agree to practice 2 highly effective contraceptive measures and must agree not to become pregnant or father a child while receiving any study therapy and for at least 3 months after completion of treatment.

**Exclusion criteria**

Patients who meet any of the following exclusion criteria cannot be included in this study:

1. Hypersensitivity to fluoroquinolones or fosfomycin.
2. Treatment with broad-spectrum antimicrobial therapy within 4 weeks of the first study treatment.
3. Intensive chemotherapy or prior hematopoietic stem cell transplantation. Treatment with hydroxyurea or corticosteroids used to control white blood cell count is allowed.
4. Fever of infectious origin or documented infection within 4 weeks of the first study treatment.
5. Presence of any serious psychiatric illness or physical condition that, in the judgment of the physicians, contraindicates the patient’s inclusion in the clinical trial.

### Who will take informed consent? {26a}

Principal investigators in each site will provide informed consent to patient candidates for participation. The latest version approved of the patient’s information sheet will be signed by all selected subjects before inclusion in the clinical trial.

In the case of selected subjects who cannot understand the study or do not have the ability to read or write, the informed consent must be provided by the legal representative before any procedure of the study is carried out.

### Additional consent provisions for collection and use of participant data and biological specimens {26b}

This study complies with the current regulations of Law 14/2007 on biomedical research regarding the protection of the rights of patients who freely wish to participate and the handling of biological samples.

No biological samples from patients will be stored after the end of the clinical trial.

## Interventions

### Explanation for the choice of comparators {6b}

#### Intervention description {11a}

The description of the interventions and comparators is detailed in Table 1.

**Table 1:** Intervention description

Groups	Interventions
Experimental group	Patients in this arm will receive study medication fosfomycin 500 mg every 8 h orally or intravenously in case of intolerance to the oral route
Control group	Ciprofloxacin 500 mg every 12 h orally or intravenously in case of intolerance to the oral route (https://clinicaltrials.gov/study/NCT01371656?term=ACCL0934&rank=1)

### Criteria for discontinuing or modifying allocated interventions {11b}

Treatment will be interrupted when any of the following events occurs first:

1. Febrile neutropenia requiring antibacterial treatment (main end point of the study).
2. Absolute neutrophil count (ANC) > 0.5 × 10^9^/L.
3. If the subject fails to achieve more than 0.5 × 10^9^/L neutrophils, treatment will end when more than 60 days have elapsed since the onset of neutropenia or on the first day of the next chemotherapy cycle (whichever occurs first).
4. Death

#### Strategies to improve adherence to interventions {11c}

Study patients will be hospitalized during the period in which they are receiving the study treatment. The investigator of each site will review and record the traceability of the medication administered.

#### Relevant concomitant care permitted or prohibited during the trial {11d}

The concomitant medication recommendations listed in the technical data form for ciprofloxacin should be taken into consideration.

Special attention should be paid to drugs that prolong the QT interval (class IA and III anti-arrhythmics, tricyclic antidepressants, macrolides, and antipsychotics) as well as tizanidine, methotrexate, and theophylline.

The concomitant medication recommendations listed in the technical data form for fosfomycin will be taken into consideration.

#### Provisions for post-trial care {30}

The health professionals participating in this clinical trial belong to the Spanish National Health System. These are non-commercial studies where the sponsor is a non-profit foundation, FINBA. The trial subjects do not receive any compensation.

#### Outcomes {12}

The primary endpoint is febrile neutropenia requiring antibacterial treatment.

- Fever is defined as a single oral temperature of 38.3 °C or a temperature of 38 °C sustained over a 1-h period.

If the patient is receiving any medication highly likely to induce fever or has previously received a transfusion, at least one positive culture or infectious focus will be required for fever to be attributed to infection.

When febrile neutropenia occurs and infection is suspected, prophylactic antibiotics will be discontinued and evaluation and therapy will be administered according to standard and local guidelines.

Secondary endpoints are as follows:

1. Documented infections. They will be classified according to Han’s criteria in one of these categories: microbiologically documented infection (bacterial viral or fungal), mixed infection, clinically documented infection, and possible infection (fever of unknown origin).
2. The amount of concomitant antibiotics prescribed in addition to prophylactic drugs will be measured as a ratio of antibiotic days per days of hospitalization. Antibiotics will be classified according to the watch/reserve classification (21).
3. Overall survival will be defined as the time from the day of randomization to the date of death (whatever the cause of death) or to the completion of the study. Fever-free survival will also be determined.

Safety will be assessed by the incidence, severity and type of adverse events (AEs), and by the microbiological assessments:

## Microbiological assessments

- Rate of patients colonized by GNMRB as determined by surveillance and metagenomic sequencing.
- Changes in the gut microbiome produced in both groups.

### Participant timeline {13}

The flow chart for the study and the schedule visits and assessments at different points in the study protocol are described in Table 2.

**Table 2:** Schedule of visits and assessments at different points of the study protocol

Procedures	Screening visit	Day^a^ 1	Day 8 ± 1	Day 15 ± 2	Day 22 ± 2	Day 29 ± 2	End of treatment
Informed consent form	X						
Inclusion/exclusion criteria	X						
Randomization		X					
Demographic data	X						
Medical history	X						
Size	X						
Weight	X	X	X	X	X	X	X
Physical examination: vital signs	X	X	X	X	X	X	X
ECOG	X						X
Electrocardiogram	X	X	X	X	X	X	X
Study treatment		x	x	x	x	x	
Hemogram	X	X	X	X	X	X	X
Biochemistry	X	X	X	X	X	X	X
Coagulation	X	X	X	X	X	X	X
Pregnancy test	X						
Concomitant medication/antimicrobials	X	X	X	X	X	X	X
Adverse effects		X	X	X	X	X	X
Microbiological culture	X	X	X	X	X	X	X
Stool sample for microbiome analysis	X						X^b^

^a^When less than 72 h have elapsed between the screening visit and day 1, it will not be necessary to repeat the electrocardiogram or laboratory studies, including microbiological studies

^b^In case the patient is unable to collect stool before receiving the first dose of empirical antibacterials, a rectal exudate will be taken with a swab

### Sample size {14}

The reviewed clinical trials and meta-analyses have consistently shown that 65% of patients receiving prophylactic the fluoroquinolones levofloxacin and ciprofloxacin will develop infectious fever (2,17,18) which is consistent with success rate of 35%. Considering that the actual rate of resistance to fluoroquinolones is established to be around 50%, we anticipate a success rate of 17% and 30% in the active control and active and experimental control arms, respectively.

If a non-inferiority margin of 5% is defined, one hundred and thirty-four patients (67 in each arm) will be needed to demonstrate non-inferiority of fosfomycin versus ciprofloxacin with 80% potency and an alpha level of 0.05.

Considering that 15% of the enrolled patients will be ineligible or unable to be evaluated, the maximum number of study participants will be 156: 78 patients in each arm.

### Recruitment {15}

There were strategies for achieving adequate participant enrolment to reach the target sample size.

Patients who are undergoing HSCT will be previously evaluated in the pre-transplant consultation while patients diagnosed with acute leukemia will be evaluated during the first days of hospitalization. Based on whether they meet the selection criteria of the FOVOCIP study protocol, they will be asked to participate in the study by signing the informed consent form.

## Assignment of interventions: allocation

### Sequence generation {16a}

The randomization will be carried out by the electronic clinical registry (eCRF) called MACRO. The management of the electronic tool is carried out by the SCReN clinical research platform work-package.

After inclusion, the randomization list included in the eCRF will automatically assign the case to the control or experimental group in 1:1 ratio. Candidates are stratified according to their baseline disease: acute leukemia or HSCT.

### Concealment mechanism {16b}

Allocation concealment will be ensured as MACRO will not release the randomization code until the patient has been recruited into the trial.

### Implementation {16c}

The patient assignment codes are made up of 4 digits. On the one hand is the hospital number, University Hospital Central of Asturias (number 01), Hospital San Pedro Alcantara (number 2), University Hospital Morales Messeguer (number 03), University Hospital La Paz (number 04), University Hospital Fundación Jimenez Diaz (number 05), University Hospital Clínico San Carlos (number 06), University Clinic Hospital of Valencia (number 07), University Hospital La Fe (number 08), University Hospital of Málaga (number 09), University Hospital Lozano Blesa of Zaragoza (number 10), and University Hospital of Burgos (number 11), and on the other hand is the consecutive number of the new patient included.

These codes are assigned by the CRF of the study.

## Assignment of interventions: blinding

### Who will be blinded {17a}

The study is open-label. The investigators and the patient are aware of the treatment administered. The project manager only knows the randomization list, so the investigator does not know the treatment that will be assigned to the patient until the candidate is randomized. The assessors and data analysts will be blinded.

### Procedure for unblinding if needed {17b}

The design is open label with only outcome assessors being blinded, so unblinding will not occur.

## Data collection and management

### Plans for assessment and collection of outcomes {18a}

To evaluate the primary and secondary variables of the study, vital signs (blood pressure, heart rate, temperature) will be checked during the physical examination at successive visits. Laboratory tests including blood count, biochemistry and coagulation, electrocardiogram (ECG), microbiological studies, and review of treatment will be performed.

### Plans to promote participant retention and complete follow-up {18b}

An end-of-treatment visit is made to coincide with the last dose of study medication (fosfomycin or ciprofloxacin). And a follow-up visit is scheduled 15 days after the last dose of treatment to perform physical examinations and record adverse effects, ECOG, concomitant treatment, electrocardiogram (ECG), laboratory investigations, and microbiological studies.

If the patient is unable to attend the follow-up visit, this can be done by telephone.

### Data management {19}

The MACRO® software is a data collection and study management application that uses a secure web browser. It provides access to clinical study data and management of the clinical study process.

The objective is that, at the end of the study, all the available forms are as follows:

- ✓ Completed by the investigator
- ✓ With no queries pending to be solved by the Investigators
- ✓ With no queries pending to be closed by the CRAs/PMs
- ✓ Verified by the CRAs
- ✓ Locked by the PMs

### Confidentiality {27}

Personal data will be processed in accordance with Regulation (EU) 2016/679 of the European Parliament and of the Council of 27 April 2016 on the protection of individuals regarding the processing of personal data and on the free movement of such data and the relevant local laws.

The data collected for the study will be identified by an alphanumeric code in such a way that it will not be possible to identify the patient. Only the investigator and authorized persons involved in the study will have access to this code and use this information exclusively for the purposes of the study. All the generated data will be recorded in the eCRF in an anonymized form.

Members of the Clinical Research Ethics Committee or Health Authorities may have access to this information in compliance with legal requirements. The confidentiality of this data will be preserved, and it will not be linked to personal data, even if the results of the study are published.

### Plans for collection, laboratory evaluation, and storage of biological specimens for genetic or molecular analysis in this trial/future use {33}

All study subjects will be screened for MDRGN gut colonization by microbiological culture at the first visit and weekly thereafter during the neutropenic period, until study completion. In each participating center, rectal exudates will be collected in ESwab™ (Copan Diagnostics Inc., Murrieta, CA) that will be sent to the microbiology laboratories for culture in selective media for MDRGN, according to the recommendations of the Spanish Society for Infectious Diseases and Clinical Microbiology (SEIMC) (22,23), specifically in CHROMagar™ *Acinetobacter* (Biomerieux, Marcy l’Etoile, France), CHROMagar™ *P. aeruginosa* (Biomerieux), CHROMagar ™ ESBL (Biomerieux), and CHROMagar™ CARBA SMART (Biomerieux) and in Columbia Agar plate (Biomerieux) as a growth and sample quality control. The isolates recovered in the selective media will be identified by MALDI-TOF/MS (Bruker Daltonics, Bremen, Germany), and the antimicrobial susceptibility testing will be performed using the Microscan system (Beckman Coulter, Brea, CA) and interpreted according to the European Committee on Antimicrobial Susceptibility Testing (EUCAST) guidelines. Bacterial samples and isolates will be stored at – 80 °C.

The clinical trial includes a substudy in a population of 30 subjects, representative of the two treatment arms, both colonized and non-colonized according to the conventional microbiological techniques results. Two samples from each of these subjects will be recovered between those processed for culture (stored at – 80 °C) at the baseline visit and at the end of the study. In these samples, the evolution of the resistome will be investigated using “shotgun” metagenomic sequencing techniques. These results will be compared with those obtained by culture.

The patient’s microbiome evolution will be studied by metataxonomic sequencing. For this purpose, a fecal sample will be collected from each study subject at the baseline visit and at the end of the study.

**Fig. 1:**
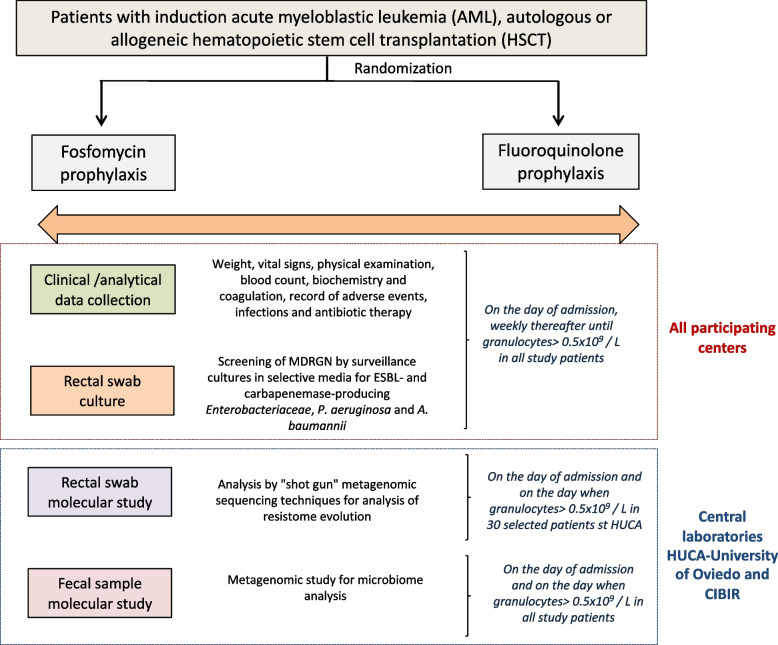
Clinical and microbiological study planning

Figure 1 shows schematically the clinical and microbiological study planning.

## Statistical methods

### Statistical methods for primary and secondary outcomes {20a}

This phase 3 trial will compare the efficacy of fosfomycin with that of ciprofloxacin in preventing the occurrence of at least one episode of neutropenic fever of infectious origin in a randomized non-inferiority design.

The primary endpoint is febrile neutropenia of infectious origin. This analysis will be performed in the intention-to-treat (ITT) and in the per-protocol population (PP). The ITT population is defined as all patients who are eligible and randomized. The PP population consists of all patients who do not have major protocol deviations, as determined by the study PI, and have received prophylactic drugs for at least of 24 h.

The rate of febrile neutropenia will be compared between the 2 arms with a chi-square test with 2-sided alpha of 0.05. The difference in the proportion and the associated 95% CIs will be provided. If, within either patient arm, the data suggest a possible difference in baseline incidence between the randomization strata, we will consider Cochran-Mantel–Haenszel test to examine the association between the incidence of febrile neutropenia and the study treatment with adjustment for the randomization strata. Logistic regression models will be performed to evaluate the treatment effect on the incidence of febrile neutropenia adjusting for stratification and other baseline patient characteristics.

The analysis of the secondary endpoints will be performed as follows:

1. Rate and type of documented infections with 2-sided alpha of 0.05. Again, logistic regression models will be performed to evaluate the treatment effect on the incidence of each event adjusting for stratification and other baseline patient’s characteristics.
2. Amount of concomitant antibiotic utilization. The analysis will be performed with a 2-sample *t*-test or the Mann–Whitney *U* test if the distribution is non-normal.
3. Survival will be analyzed using the Kaplan–Meier estimate. The K-M survival curves and medians (if estimable) along with their 2-sided 95% CIs will be provided for each treatment arm. The log-rank test will be used to compare treatment arms. Differences in survival without fever will also be assessed with a log-rank test at the 5% significance level.
4. Adverse events. This end point is included in the safety analysis and will be performed in the SP. AE will be categorized as severe and non-severe, as this classification determines the notification procedure. Intensity of AE will be evaluated according to the National Common Terminology Criteria for Adverse Events (NCI CTCAE), version 5.034.

The microbiological safety assessments will be analyzed as specified in item 33.

### Interim analyses {21b}

An interim analysis will be performed by a data and safety monitoring board (DSMB) for the primary outcome measure after the inclusion of 50 patients. O’Brien- Fleming stopping criteria will be used for the primary outcome (24). If the *p* value is lower than 0.0107 for inferiority of fosfomycin to ciprofloxacin at the 5% margin, the DMSC may recommend early termination of the trial. Accrual will not be suspended for these monitoring and analysis.

### Methods for additional analyses (e.g., subgroup analyses) {20b}

There is no planned sub-group analysis.

### Methods in analysis to handle protocol non-adherence and any statistical methods to handle missing data {20c}

To prevent attrition bias, two types of analysis will be performed: an intention-to-treat analysis, in which all randomized patients will be analyzed according to the group to which they were randomized, regardless they received the assigned treatment or not, and, in addition, a per-protocol analysis will be performed in all randomized patients who finally received the assigned treatment for at least of 24 h (2 doses of ciprofloxacin and 3 doses of fosfomycin).

The imputation of missing variables will consider the distribution of the variables, the allocation group, the center, and the baseline condition (acute leukemia or stem cell transplant).

The method of fully conditional specification (FCS) using the MI procedure of SAS, version 9.4 (SAS Inc, Cary, NC, USA), will be used [15]. In this method, FCS imputations are generated sequentially by specifying an imputation model for each variable given the other variables. Ten imputations will be used in the imputation model and sensitivity analysis will be performed.

### Plans to give access to the full protocol, participant-level data, and statistical code {31c}

Dissemination of results directed to patients will be channeled through the Spanish Agency for Medicines and Health Products, and their content will be adapted to patients.

## Oversight and monitoring

### Composition of the coordinating center and trial steering committee {5d}

The *steering committee (SC)* will be the ultimate decision-making body of the FOVOCIP consortium and will be composed of the project coordinators (Teresa Bernal and Javier Fernandez) plus one representative of each participating institution. SC will meet at least once a year. Coordinators will chair SC meetings. Decision-making will be made based on consensus.

The *executive board* (EB) will be responsible for the overall strategic and technical management and will report directly to the SC. The EB will be comprised of the project coordinators plus 2 additional PIs of different centers participating in the study (elected by voting in the first SC meeting) plus the project monitor (PM) and the project assistant (PA). EB will execute the decisions of the SC as well as monitor the effective and efficient implementation of the project. Minutes of the EB meetings, once accepted, will be sent to the SC members in order to keep them informed. The coordinators will chair the EB meetings. The EB will meet at least quarterly and upon justified written request of any member of the EB.

*The management team* will support the PI and coPI coordinators in the day-to-day operational management and fulfilling the project’s administration tasks. The management team will comprise the PM of the IDIVAL and a full-time project assistant (PA) employed by the research team.

Finally, PIs in each center are responsible for including identifying potential recruits and taking consent.

### Composition of the data monitoring committee, its role and reporting structure {21a}

A meeting will be held by non-study personnel to evaluate the results of 80 patients. The interim analysis committee will be composed of a hematologist, a microbiologist and a clinical pharmacologist.

### Adverse event reporting and harms {22}

The investigators will be responsible for collecting all the adverse events in the clinical history based on those referred by the patient spontaneously or by interview during the follow-up visits. The causality of the adverse event related to the intervention will be evaluated and recorded in the clinical history and in the eCRF.

The investigators will report to the sponsor all serious adverse events occurring to subjects treated in the clinical trial, without undue delay, but not later than within 24 h of obtaining knowledge of the events.

The sponsor will report to the Spanish Agency for Medicines and Health Products all relevant information about suspected unexpected serious adverse reactions to investigational medicinal products occurring in this clinical trial.

### Frequency and plans for auditing trial conduct {23}

The CRA is a person independent of the research team who is not involved in patient recruitment and follow-up.

The local CRA makes a start-up and closing visit to each site. A first visit will take place in each center 30 days after the inclusion of the first patient. Subsequently, 4 to 6 visits will be performed per site, with a frequency of 1 visit every 4 months.

During the monitoring visits, 100% of the informed consents form, selection criteria, events and adverse reactions, and main and secondary variables (neutrophil counts, fever, study treatment, stored samples) will be reviewed. The remaining the variables will be reviewed every 5 patients.

### Plans for communicating important protocol amendments to relevant parties (e.g., trial participants, ethical committees) {25}

All notifications defined as relevant or non-relevant amendments will be made to the Spanish Agency for Medicines and Health Products and to the Ethics Committee of reference in accordance with Spanish legislation on clinical trials RD 1090/2015 of December 4. These decrees regulate clinical trials with medicinal products: the Ethics Committees for Research with medicines and the Spanish Registry of Clinical Trials.

### Dissemination plans {31a}

The results of the present project will be communicated to the scientific community through scientific publications. All publications will be open access. It is foreseen that the project will result in at least 2 peer reviews. All generated data will be accessible and stored in open access data repositories. A list of priority conferences to be targeted will be developed, aiming to achieve local, national, and international impact.

## Discussion

The debate on the efficacy of FQ prophylaxis in the era of multidrug resistance is ongoing. Our study will contribute to this debate by comparing the efficacy and safety of ciprofloxacin with fosfomycin in a homogeneous population of patients treated at several centers with different prevalence in MRGNB.

The supporters of FQ prophylaxis (either ciprofloxacin or levofloxacin) have reported an increase in the incidence of Gram-negative bacterial infections [16] and even mortality [17], when the fluoroquinolone prophylaxis program was stopped. To avoid this unethical risk of mortality, the study was design using ciprofloxacin as an active control instead of placebo. The selection of fosfomycin was based not only on its favorable pharmacological profile but also on retrospective data suggesting that it might be an effective prophylactic drug in hematological patients [18].

The opponents of FQ prophylaxis argue that these drugs are associated with gut colonization by MRGNB. In hematological patients, gut colonization is associated with blood stream infections when neutropenia occurs [5]. Our study also addresses this important question. First, the planned weekly surveillance cultures will provide information about the days of exposure to ciprofloxacin that are needed until gut colonization occurs and the way in which the patient’s characteristics, the chemotherapy administered, and the exposure to ciprofloxacin interact with each other and modulate the colonization. Second, we will analyze if there is any difference in the risk of colonization in both treatment arms. Finally, we will address the microbiological safety of ciprofloxacin and fosfomycin through a sequencing approach. On one side, we plan to perform metagenomic sequencing and resistome analysis to detect subclinical gut colonization. It has been documented a low sensitivity of culture-based methods aimed to surveillance of MRGNB carriers [19]. This could be more frequent at the beginning of colonization, when the proportion of multi-resistant bacteria in the microbiota is still a minority. Under-detected colonization could have epidemiological implications, since these patients would not be subjected to contact precautions despite being potentially sources of transmission of MRGNB, and clinical repercussions, since the colonized patients could suffer bacteremia or other infections by these strains. On the other side, metataxonomic sequencing will help to evaluate prospectively the changes in patients’ microbiome when they receive FQ prophylaxis, changes that we expect to be more pronounced than those occurring under fosfomycin prophylaxis.

Our study will have some limitations: first it will be open, which might favor the early termination of prophylaxis in the fosfomycin arm. However, a strict definition of febrile neutropenia of infectious origin has been specified in the protocol, which excludes transfusional associated fever or fever related to cytarabine (a common chemotherapy drug used in acute myeloid leukemia) or other drugs. Second, this is not a superiority trial, but a non-inferiority one, because it would have been unethical to conduct a trial comparing fosfomycin with placebo. Notwithstanding the limitations of this type of design [20], if fosfomycin proves to be equally effective and safer than ciprofloxacin, it will open the possibility of having more than one family of prophylactic drugs, making it possible to reduce the resistance associated with continued exposure to the same antibiotic and to select one treatment or another based on bacterial ecology and local resistance.

In summary, the present project addresses an extremely important health problem by evaluating the efficacy of fosfomycin, a broad-spectrum antibiotic with a better safety profile and a lower potential of resistance induction than ciprofloxacin, in the prevention of infections in hematological patients with high-risk neutropenia. The battery of microbiological studies that will be carried out will allow us to evaluate prospectively, for the first time, the microbiological safety of both pharmacological strategies and the selection of MRGNB in each arm and will generate valuable information on the way in which changes that occur in the fecal microbiome of the patients throughout the treatment modify the evolution of the studied diseases.

## Trial status

Current version of the protocol is 3.0 of 11 of May 2021.

Trial is currently recruiting. The first patient was included in March 2021. At the time of submission, 106 patients have been recruited. The recruitment is expected to be completed by March 2024.

## Data Availability

The protocol and the results of the trial will be the exclusive property of the sponsor, who reserves the right to request on its behalf the industrial property rights that may derive from them. There are no agreements with other entities. The principal investigators are responsible for the preparation of the final report and therefore will have access to all available data. The anonymized data and the statistical code used for the analysis will be available from the principal investigators upon reasonable request. Data obtained from genomic studies will be deposited in repositories and databases and international public platforms such as the National Center for Biotechnology Information (NCBI).

## Abbreviations

AEMPS: Spanish Agency of Medicines and Medical Devices
ANC: Absolute neutrophil count
CRA: Clinical research associate
EC: Ethics committee
ECG: Electrocardiogram
eCRF: Electronic case report form
ECOG: Eastern Cooperative Oncology Group
ESBL: Extended-spectrum B-lactamase *Enterobacteriaceae*
DSMB: Data and safety monitoring board
FINBA: Fundación para la Investigación y la innovación
FQ: Fluoroquinolones
HCTI: Hematopoietic Comorbidity Transplant Index
ITT: Intention to treat
HSCT: Hematopoietic stem cell transplant
MDRGNB: Multidrug-resistant Gram-negative bacteria
NCTCAE: National Common Terminology Criteria for Adverse Events
PA: Project assistant
PI: Principal investigator
PM: Project manager
PP: Per protocol
SCReN: Spanish Clinical Research Network

## References

[1] Freifeld AG, Bow EJ, Sepkowitz KA, Boeckh MJ, Ito JI, Mullen CA (2011). Clinical practice guideline for the use of antimicrobial agents in neutropenic patients with cancer: 2010 update by the infectious diseases society of america. Clin Infect Dis.

[2] Gafter-Gvili A, Fraser A, Paul M, Leibovici L (2005). Meta-analysis: antibiotic prophylaxis reduces mortality in neutropenic patients. Ann Intern Med.

[3] Satlin MJ, Chavda KD, Baker TM, Chen L, Shashkina E, Soave R (2018). Colonization with levofloxacin-resistant extended-spectrum β-lactamase-producing Enterobacteriaceae and risk of bacteremia in hematopoietic stem cell transplant recipients. Clin Infect Dis.

[4] Girmenia C, Rossolini GM, Piciocchi A, Bertaina A, Pisapia G, Pastore D (2015). Infections by carbapenem-resistant Klebsiella pneumoniae in SCT recipients: a nationwide retrospective survey from Italy. Bone Marrow Transplant.

[5] Ballo O, Tarazzit I, Stratmann J, Reinheimer C, Hogardt M, Wichelhaus TA (2019). Colonization with multidrug resistant organisms determines the clinical course of patients with acute myeloid leukemia undergoing intensive induction chemotherapy. PLoS ONE.

[6] Tacconelli E, Cataldo MA, Dancer SJ, De Angelis G, Falcone M, Frank U (2014). ESCMID guidelines for the management of the infection control measures to reduce transmission of multidrug-resistant Gram-negative bacteria in hospitalized patients. Clin Microbiol Infect.

[7] Castañón C, Fernández Moreno A, Fernández Verdugo AM, Fernández J, Martínez Ortega C, Alaguero M (2019). The value of adding surveillance cultures to fluoroquinolone prophylaxis in the management of multiresistant gram negative bacterial infections in acute myeloid leukemia. JCM.

[8] Torres I, Huntley D, Tormo M, Calabuig M, Hernández-Boluda JC, Terol MJ (2022). Multi-body-site colonization screening cultures for predicting multi-drug resistant Gram-negative and Gram-positive bacteremia in hematological patients. BMC Infect Dis.

[9] Gudiol C, Aguilar-Guisado M, Azanza JR, et al. Executive summary of the consensus document of the Spanish Society of Infectious Diseases and Clinical Microbiology (SEIMC), the Spanish Network for Research in Infectious Diseases (REIPI) and the Spanish Society of Haematology and Haemotherapy (SEHH) on the management of febrile neutropenia in patients with hematological malignancies. Enferm Infecc Microbiol Clin. 2020;38(4):174–81. 10.1016/j.eimc.2019.01.013 30926172

[10] Holler E, Butzhammer P, Schmid K, Hundsrucker C, Koestler J, Peter K (2014). Metagenomic analysis of the stool microbiome in patients receiving allogeneic stem cell transplantation: loss of diversity is associated with use of systemic antibiotics and more pronounced in gastrointestinal graft-versus-host disease. Biol Blood Marrow Transplant.

[11] Hakim H, Dallas R, Wolf J, Tang L, Schultz-Cherry S, Darling V (2018). Gut microbiome composition predicts infection risk during chemotherapy in children with acute lymphoblastic leukemia. Clin Infect Dis.

[12] Wojenski DJ, Barreto JN, Wolf RC, Tosh PK (2014). Cefpodoxime for antimicrobial prophylaxis in neutropenia: a retrospective case series. Clin Ther.

[13] Donnelly JP, Maschmeyer G, Daenen S (1992). Selective oral antimicrobial prophylaxis for the prevention of infection in acute leukaemia-ciprofloxacin versus co-trimoxazole plus colistin. The EORTC-Gnotobiotic Project Group. Eur J Cancer..

[14] Falagas ME, Vouloumanou EK, Samonis G, Vardakas KZ (2016). Fosfomycin. Clin Microbiol Rev.

[15] Liu Y, De A (2015). Multiple imputation by fully conditional specification for dealing with missing data in a large epidemiologic study. IJSMR.

[16] Clerici D, Galli L, Greco R, et al. Levofloxacin prophylaxis vs no prophylaxis in patients with neutropenia within an endemic country for carbapenem-resistant GNB. Blood Adv. 2023;7(9):1621–34. 10.1182/bloodadvances.2022008226 PMC10182274 36409602

[17] Reuter S, Kern WV, Sigge A, Döhner H, Marre R, Kern P (2005). Impact of fluoroquinolone prophylaxis on reduced infection-related mortality among patients with neutropenia and hematologic malignancies. Clin Infect Dis.

[18] Zapolskaya T, Perreault S, McManus D, Topal JE (2018). Utility of fosfomycin as antibacterial prophylaxis in patients with hematologic malignancies. Support Care Cancer.

[19] Ridgway JP, Peterson LR, Thomson RB, Miller BA, Wright M-O, Schora DM (2014). Sensitivity of surveillance testing for multidrug-resistant Gram-negative bacteria in the intensive care unit. J Clin Microbiol.

[20] Hahn S (2012). Understanding noninferiority trials. Korean. J Pediatr.

